# Supplementary material for: Metabolically Improved Stem Cell Derived Hepatocyte-Like Cells Support HBV Life Cycle and Are a Promising Tool for HBV Studies and Antiviral Drug Screenings
Source: Biomedicines. 2022 Jan 26;10(2):268. doi: 10.3390/biomedicines10020268 (PMC8869365; doi:10.3390/biomedicines10020268)
Supplement: Supplementary file 1 [file biomedicines-10-00268-s001.zip › biomedicines-1547268-supplementary.pdf]

Supplementary Material

Metabolically Improved Stem Cell Derived Hepatocyte-Like Cells Support HBV Life Cycle and Are a Promising Tool for HBV Studies and Antiviral Drug Screenings

Tine Tricot [1,*,†], Hendrik Jan Thibaut [2,3,*,†], Kayvan Abbasi [2], Ruben Boon [1,4], Nicky Helsen [1,5], Manoj Kumar [1], Johan Neyts [2,†] and Catherine Verfaillie [1,*,†]

[1] Stem Cell Institute, Rega Institute KU Leuven, 3000 Leuven, Belgium; ruben.boon@kuleuven.be (R.B.); nicky.helsen@ismar.com (N.H.); manoj.kumar@kuleuven.be (M.K.)

[2] Department of Microbiology, Immunology and Transplantation, Virology and Chemotherapy, Rega Institute KU Leuven, 3000 Leuven, Belgium; kayvan.abbasi@kuleuven.be (K.A.); johan.neyts@kuleuven.be (J.N.)

[3] Department of Microbiology, Immunology and Transplantation, Translational Platform Virology and Chemotherapy (TPVC), Rega Institute KU Leuven, 3000 Leuven, Belgium

[4] Laboratory for Functional Epigenetics, Department of Human Genetics, Rega Institute KU Leuven, 3000 Leuven, Belgium

[5] Ismar Healthcare NV, 2500 Lier, Belgium

* Correspondence: tine.tricot@kuleuven.be (T.T.); Hendrikjan.thibaut@kuleuven.be (H.J.T.); catherine.verfaillie@kuleuven.be (C.V.); Tel.: +32-16-37-71-09 (T.T.); +32-16-32-16-82 (H.J.T.); +32-16-37-26-54 (C.V.)

† These authors contributed equally to this work.

Supplementary Table……………………………………………………………………………1

Supplementary Figures…………………………………………………………………………2

Supplementary Tables

Table S1. Primer list.

GENE	FORWARD PRIMER	REVERSE PRIMER
RPL19	**ATTGGTCTCATTGGGGTCTAAC**	**AGTATGCTCAGGCTTCAGAAGA**
ALB	**ATGCTGAGGCAAAGGATGTC**	**AGCAGCAGCACGACAGAGTA**
AAT	**AGGGCCTGAAGCTAGTGGAT**	**TCCTCGGTGTCCTTGACTTC**
HNF4	**ACTACGGTGCCTCGAGCTGT**	**GGCACTGGTTCCTCTTGTCT**
NTCP	**ATCGTCCTCAAATCCAAACG**	**CCACATTGATGGCAGAGAGA**

Table S2. Antibody list.

ANTIBODY	CATALOG NUMBER	COMPANY	DILUTION
HNF4	**Ab41898**	**Abcam**	**1:200**
NTCP	**HPA042727**	**Sigma-Aldrich**	**1:500**
HBcAg	**B0586**	**Dako**	**1:750**
HBsAg	**SAB 4700767-100UG**	**Sigma-Aldrich**	**1:500**
ALEXA 555 DONKEY ANTI-RABBIT	**Thermo Fisher**	**A31572**	**1:500**
ALEXA 488 DONKEY ANTI-RABBIT	**Thermo Fisher**	**A21206**	**1:500**
ALEXA 555 DONKEY ANTI-MOUSE	**Thermo Fisher**	**A31570**	**1:500**
ALEXA 488 DONKEY ANTI-MOUSE	**Thermo Fisher**	**A21202**	**1:500**
ISOTYPE RABBIT IgG	**BD Pharmingen**	**550875**	**Used as control in same concentration as primary antibody**
ISOTYPE MOUSE IgG2a	**Sigma-Aldrich**	**M9144-1MG**	**Used as control in same concentration as primary antibody**

Supplementary Figures

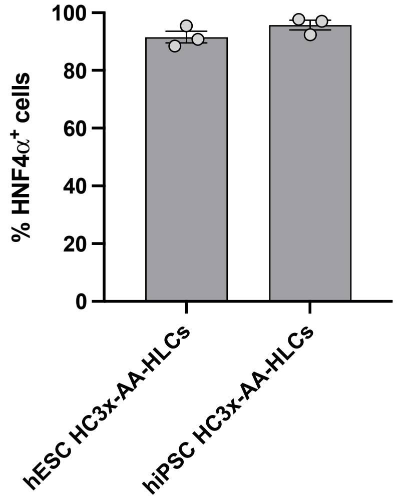

Figure S1. Purity of the hESC and hiPSC HC3x-AA-HLC differentiation. Quantification of immunofluorescence staining for HNF4α⁺ hESC and hiPSC HC3x-AA-HLCs. All data is shown as mean ± SEM.

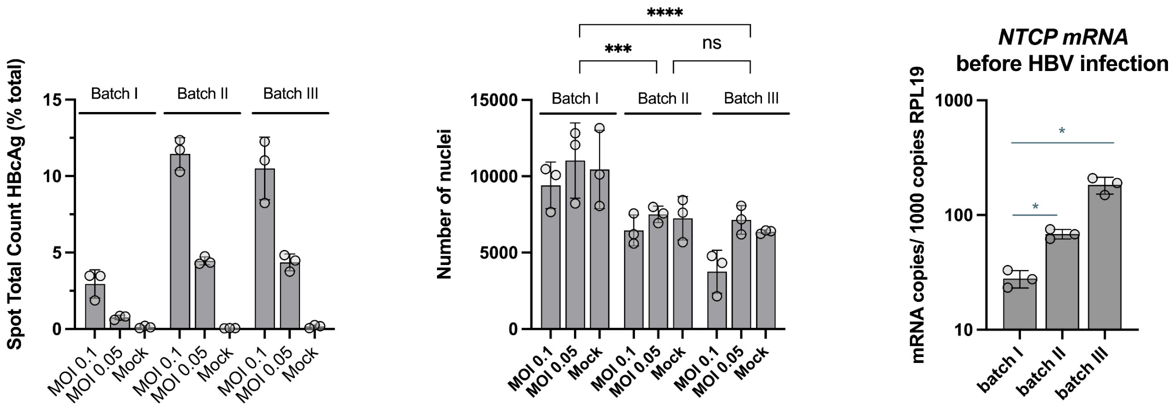

Figure S2. Variability in HBV infection efficiency is possibly caused by differences in cell density and *NTCP* expression levels. Quantification of immunofluorescence staining for HBcAg and cell number in mock and HBV-infected HC3x-AA-HLCs (MOI 0.1 and 0.05), as well as gene expression analysis for *NTCP* in 3 different experiments. *RPL19* was used for normalization. All data is shown as mean ± SEM.
